# Karyotype differentiation of four *Cestrum* species (Solanaceae) revealed by fluorescent chromosome banding and FISH

**DOI:** 10.1590/S1415-47572009000200019

**Published:** 2009-06-01

**Authors:** Thiago Fernandes, Letícia do Nascimento Andrade de Almeida Rego, Mariana Nardy, Priscila Mary Yuyama, André Luís Laforga Vanzela

**Affiliations:** Laboratório de Biodiversidade e Restauração de Ecossistemas, Centro de Ciências Biológicas, Universidade Estadual de Londrina, Londrina, PRBrazil

**Keywords:** * Cestrum*, chromosome banding, FISH, heterochromatin, physical maps, 45S and 5S rDNA

## Abstract

The karyotypes of four South American species of *Cestrum* (*C. capsulare,C. corymbosum,C. laevigatum* and *C. megalophylum*) were studied using conventional staining, C-CMA/DAPI chromosome banding and FISH with 45S and 5S rDNA probes. The karyotypes showed a chromosome number of 2*n* = 2*x* = 16, with metacentric chromosomes, except for the eighth submeta- to acrocentric pair. Several types of heterochromatin were detected, which varied in size, number, distribution and base composition. The C-CMA^+^ bands and 45S rDNA were located predominantly in terminal regions. The C-CMA ^+^ /DAPI ^+^ bands appeared in interstitial and terminal regions, and the C-DAPI ^+^ bands were found in all chromosome regions. The 5S rDNA sites were observed on the long arm of pair 8 in all species except *C. capsulare*, where they were found in the paracentromeric region of the long arm of pair 4. The differences in band patterns among the species studied here, along with data from other nine species reported in the literature, suggest that the bands are dispersed in an equilocal and non-equilocal manner and that structural rearrangements can be responsible for internal karyotype diversification. However, it is important to point out that the structural changes involving repetitive segments did not culminate in substantial changes in the general karyotype structure concerning chromosome size and morphology.

## Introduction

The genera *Cestrum* L., *Sessea* Ruiz & Pav. and *Vestia* Willd. belong to the tribe Cestreae. Their representatives are trees, subtrees or bushes that are distributed in tropical and subtropical America (Hunziker, 1979). The few *Cestrum* species that have been cytogenetically studied presented the largest chromosomes of the family, up to about 14 μm long (Fregonezi *et al.*, 2006; Las Peñas *et al.*, 2006). *Cestrum* is the largest genus of the tribe (Judd *et al.*, 1999), with 50 species in Brazil (Hunziker, 2001). Chromosome banding studies have shown that in *Cestrum* heterochromatin varies in amount and type, and that the similarity observed among karyotypes regarding chromosome size and morphology is apparent (see Berg and Greilhuber, 1992, 1993a, 1993b; Fregonezi *et al.*, 2006). In spite of the small number of species studied*,* significant information about the occurrence and distribution of different classes of repetitive DNA (45S and 5S rDNA, microsatellites and retroelements) has been reported (Berg and Greilhuber, 1992, 1993a, 1993b; Fregonezi *et al.*, 2006, 2007; Las Peñas *et al.*, 2006). Sykorová *et al.* (2003a, 2003b) described the absence of *Arabidopsis* (DC.) Heynh.-type (TTTAGGG)_n_ telomeres, which seem to be replaced by an A/T-rich minisatellite family in *Cestrum*, *Sessea* and *Vestia*, besides an uncommon occurrence of 45S and 5S rDNA in B-chromosomes of *Cestrum parqui* L'Her. In two other studies, Fregonezi *et al.* (2006, 2007) described different numbers of 45S rDNA sites and the constancy in number and location of 5S rDNA sites in four species of *Cestrum*, plus the occurrence of a *Ty*3-*gypsy* retrotransposon family dispersed along chromosomes and forming blocks associated with NORs in *C. intermedium* Sendtn. and *C. strigilatum* Ruiz & Pav.

The aim of this study was to extend the information about the occurrence and the physical location of some repetitive DNAs in four South American *Cestrum* species. This work involved conventional karyotype determination and the physical location of different heterochromatin families, as well as the 45S and 5S rDNA sites. Such information is useful in evolutionary studies of this group and allows the development of new directions in the elucidation of karyotype differentiation in *Cestrum*.

**Figure 1:**
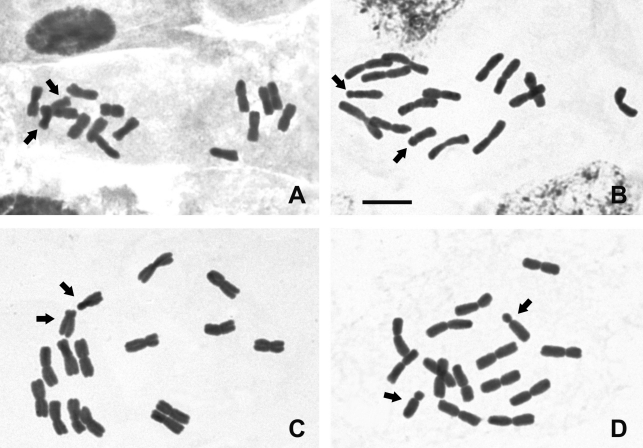
Conventional staining in *Cestrum* species (2*n* = 16): *C. capsulare* (A), *C. corymbosum* (B), *C. laevigatum* (C) and *C. megalophylum* (D). Arrows point to submeta-to-acrocentric pair 8 and some chromocenters in partial nuclei of *C. corymbosum*. Bar = 10 μm.

**Figure 2:**
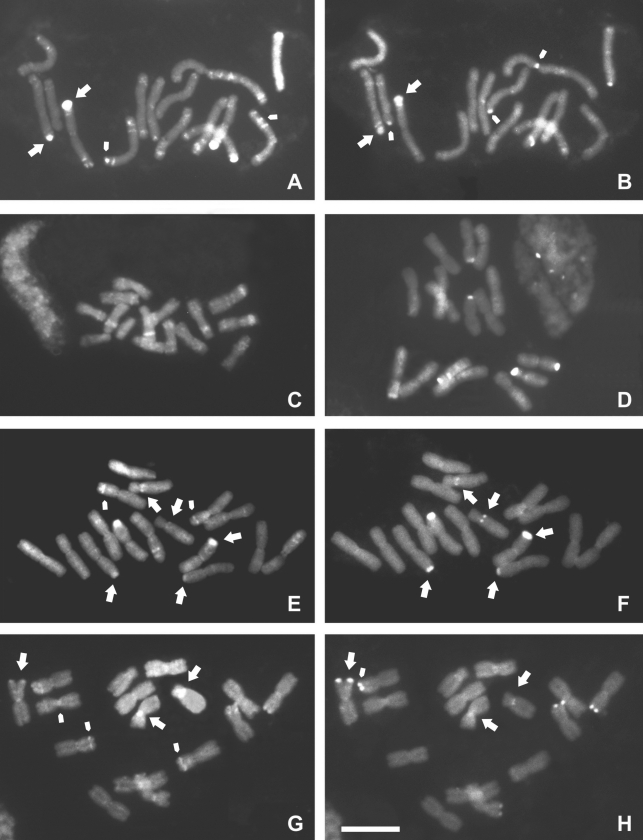
Chromosome banding with fluorochromes in *Cestrum* species: C-DAPI banding in *C. capsulare* (A); *C. corymbosum*, incomplete metaphase (C); *C. laevigatum* (E); and *C. megalophylum* (G). C-CMA banding in *C. capsulare* (B), *C. corymbosum* (D), *C. laevigatum* (F) and *C. megalophylum* (H). The larger arrows indicate C- CMA^+^/DAPI^+^ bands; the smaller arrows indicate C-CMA^-^/DAPI^+^ or C-CMA^+^/DAPI^-^ bands. Bar = 10 μm.

**Figure 3:**
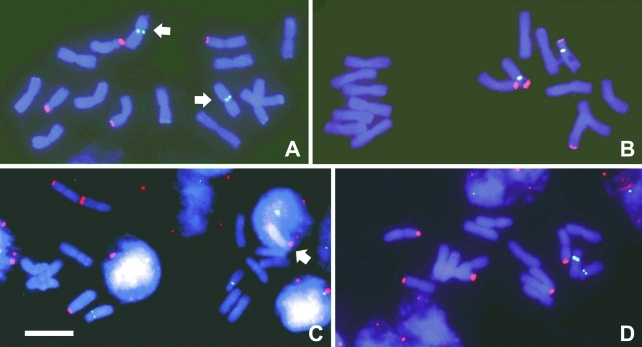
FISH with the 45S rDNA probe detected with anti-digoxigenin-rhodamine (red) and the 5S rDNA probe detected with biotin-FITC (green) in *Cestrum* species (2*n* = 16): (A) *Cestrum capsulare*, (B) *C. corymbosum*, and (C) *C. laevigatum*. Note the four 45S rDNA sites always located in terminal regions and two 5S rDNA sites always in proximal regions of pair 8, except for *Cestrum capsulare*, where the 5S rDNA signal was observed on the metacentric pair 4 (arrows). In Figure (C), the arrow points to a chromosome containing the 45S rDNA site overlapped in the nuclei. (D) *C. megalophylum* showing five terminal signals of 45S rDNA and two proximal signals of 5S rDNA on pair 8. Bar = 10 μm.

**Figure 4:**
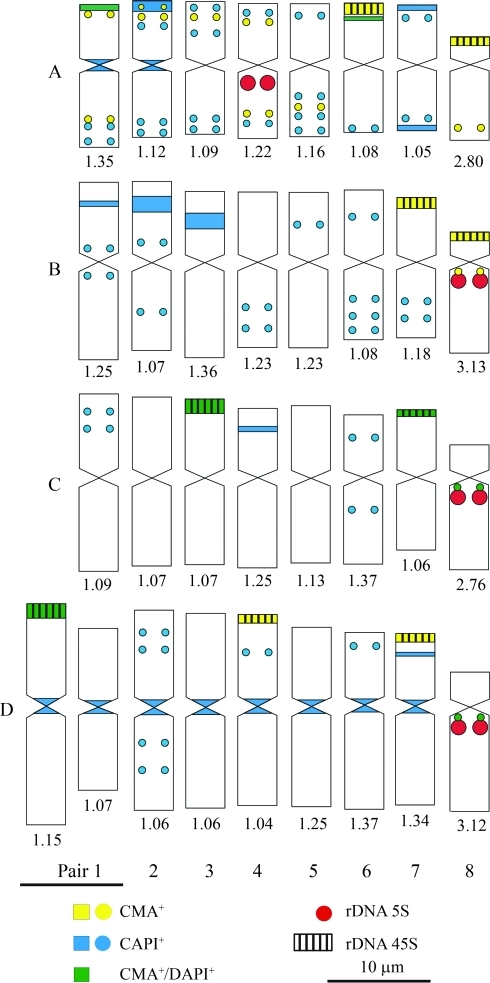
Idiograms with physical location of repetitive segments in *Cestrum capsulare* (A), *C. corymbosum* (B), *C. laevigatum* (C) and *C. megalophylum* (D). Numbers below the idiograms correspond to arm ratios, according to Guerra (1986): metacentric (m) with arm ratios (*ar*) from 1.00 to 1.49, submetacentric (sm) with *ar* from 1.50 to 2.99, acrocentric (a) with *ar* > 3.0.

## Materials and Methods

Seeds of four *Cestrum* species were collected in five different localities, always from three individuals of each population, which included (i) *C. capsulare* Carvalho & Schnoor from Curiúva and Ventania, Paraná State, Brazil (24° 03'14” S, 50° 27'23” W), (ii) *C. corymbosum* Schltdl. from Tibagi, Paraná State, Brazil (24° 38' 44” S, 50° 34' 11” W), (iii) *C. laevigatum* Schltdl. from Misiones, Argentina (26° 48' 25” S, 54° 44' 22” W), and (iv) *C. megalophylum* Dunal from Salvador, Bahia State, Brazil (12° 52' 51” S, 38° 21' 41” W). Seeds were germinated and the seedlings were cultivated in the Laboratory of Biodiversity and Restoration of Ecosystems (LABRE) at the State University of Londrina (UEL). Vouchers are kept at the FUEL herbarium. For cytogenetic analysis, slides were prepared from root tips pretreated with 0.05% colchicine for 4 h, fixed in Farmer solution (ethanol/acetic acid 3:1, v:v) for up to 24 h, and stored at -20 °C. Roots were hydrolyzed in 1 M HCl for 10 min at 60 °C, dissected in a drop of 45% acetic acid and coverslipped. Coverslips were removed in liquid nitrogen, and the preparations were stained with 2% Giemsa and permanently mounted in Entellan (Merck). Chromosome measurements were made based on five metaphases, using the MicroMeasure 3.3 software (http://www.biology.colostate.edu), and the values of total length, arm ratio and relative size were considered for idiogram construction. Chromosomes were classified according to Guerra (1986).

Chromosome banding was performed as described by Schwarzacher *et al.* (1980), with minor modifications. Root tips were digested in an enzyme solution composed of 4% cellulase (w/v) and 40% pectinase (v/v) at 37 °C and dissected in a drop of 45% acetic acid. The slides were aged for three days and incubated in 45% acetic acid for 10 min at 60 °C, in 5% barium hydroxide for 10 min at room temperature, and in 2xSSC, pH 7.0, for 10 min at 60 °C. The samples were then washed in distilled water, air dried and stained with fluorochromes: 0.5 mg/mL chromomycin A3 (CMA) for 1.5 h, and 2 μg/mL 4-6-diamidino-2-phenylindole (DAPI) for 30 min. Slides were mounted with a medium composed of glycerol/McIlvaine buffer (pH 7.0) 1:1, plus 2.5 mM MgCl_2_. The option for CMA/DAPI staining after chromosome banding was to improve the contrast of the fine bands, due to greater retreat of the euchromatin and preservation of the heterochromatin.

Fluorescent *in situ* hybridization (FISH) was performed according to the procedures described by Heslop-Harrison *et al.* (1991) and Cuadrado and Jouve (1994), with minor modifications. Slides were prepared as described for chromosome banding and immediately used for FISH. A wheat p*Ta*71 probe containing the 45S rDNA sequence (Gerlach and Bedbrook, 1979) was labeled with digoxigenin-11-dUTP by nick translation, and a p*Ta*794 probe containing the 5S rDNA sequence (Gerlach and Dyer, 1980) was labeled with biotin-14-dATP, also by nick translation. For each slide 34 μL of a denatured hybridization mixture was applied. This mixture was composed for 100 ng of labeled probe (4 μL of each probe), 10 0% formamide (15 μL), 50% polyethylene glycol (6 μL), 20xSSC (3 μL), 100 ng of calf thymus DNA (1 μL), and 10% SDS (1 μL). Both slide and mixture were denatured at 90 °C for 10 min, and hybridization was performed overnight at 37 °C in a humidified chamber. Post-hybridization washes were carried out in 2xSSC, 20% formamide in 0.1xSSC, 0.1xSSC and 4xSSC/0.2% Tween 20, all at 42 °C. The probes were simultaneously detected with a solution composed of 5% BSA, avidin-FITC conjugate and antidigoxigenin-rhodamine conjugate (100:1:1, v:v:v), followed by post-detection baths in 4xSSC/0.2% Tween 20 at room temperature. Slides were mounted in 25 μL of a medium composed of 23 μL of DABCO solution [1,4-diaza- bicyclo (2.2.2)-octane (2.3%), 20 mM Tris HCl, pH 8.0, (2%) and glycerol (90%), in distilled water], 1 μL of 2 μg/mL DAPI, and 1 μL of 50 mM MgCl_2_.

All the images were acquired with a Leica DM 4500 B microscope equipped with a DFC 300FX camera and the Leica IM50 4.0 software, and optimized for best constrast and brightness with iGrafx Image software.

## Results

Conventional karyotype analysis of the four *Cestrum* species showed a constant chromosome number of 2*n* = 2*x* = 16 with metacentric chromosomes, except for the smallest pair that was from submetacentric to acrocentric (Figures 1A, C and D Figure 4). The interphase nuclei were also always of the reticulate type, without evident chromocenters when the nuclei were more condensed (Figure 1A) and with some chromocenters when the nuclei were decondensed (Figure 1B). Differences were found in the karyotype formulae and haploid set sizes, being 7 m + 1 sm and 24 μm in *Cestrum capsulare* (Figures 1A and 4A), 7 m + 1 a in *C. corymbosum*, and 7 m + 1 sm in *C. laevigatum*, but both species presented 28 μm of haploid set sizes (Figures 1B, 4B and Figures 1C and 4C, respectively). An atypical idiogram was obtained for *C. megalophylum*, due to the presence of the heteromorphic pair 1 (Figures 1D and 4D), but the karyotype formula was 7 m + 1 a. The results of the chromosome measurements showed that *C. megalophylum* has the largest and *C. capsulare* the smallest haploid set size, while *C. corymbosum* and *C. laevigatum* showed similar karyotype length.

CMA/DAPI staining after treatment with acetic acid, barium hydroxide and 2xSSC was useful to reveal CMA^+^/DAPI^-^, CMA^-^/DAPI^+^ and CMA^+^/DAPI^+^ bands (Figure 2). Chromosome banding demonstrated quantitative and qualitative variation among the four species studied. The larger heterochromatic blocks were located preferentially in the terminal and subterminal regions. Most of the smaller blocks or dots were observed in the interstitial regions (Figure 2), except for *Cestrum capsulare* that exhibited a great number of C-DAPI^+^ dots located in interstitial and terminal regions (Figure 4A). *Cestrum corymbosum* showed the largest C-DAPI^+^ bands in the interstitial region of the short arm of pairs 1, 2 and 3, and dots in the interstitial regions of pairs 1, 2, 4, 5, 6 and 7 (Figures 2C and 4B). *Cestrum laevigatum* presented the smallest number of C-DAPI^+^ bands, located mainly in the interstitial regions of the short arms of pairs 1, 4 and 6. The only paracentromeric band was C-CMA^+^/DAPI^+^, observed on the long arm of pair 8 (Figures 2E and 4C). *Cestrum megalophylum* showed pericentromeric C-DAPI^+^ blocks on all chromosomes, except for pair 8, which was C-CMA^+^/DAPI^+^. It is important to point out that pericentromeric C-DAPI^+^ bands were not strongly evident in all the preparations of *C. megalophylum.* Larger interstitial C-DAPI^+^ bands were found in pairs 3 and 7, besides interstitial dots in pairs 2, 4 and 6 (Figures 2G and 4D).

C-CMA^+^ bands were usually present in the terminal regions of the short arms of meta-, submeta- and acrocentric chromosomes. In *C. capsulare*, besides the terminal blocks on pairs 6 and 8 associated with NORs, some interstitial dots were found in subterminal regions, except in pairs 6 and 7. C-CMA^+^/DAPI^+^ bands were observed in the terminal region of pair 1 and in interstitial positions on the short arms of pair 6 (Figures 2B and 4A). *Cestrum corymbosum* showed terminal C-CMA^+^ blocks on pairs 7 and 8, in addition to a paracentromeric dot on the long arm of pair 8 (Figures 2D and 4B). In *Cestrum laevigatum,* C-CMA^+^/DAPI^+^ blocks were observed in the terminal region of pairs 3 and 7, besides a paracentromeric dot on the long arm of pair 8 (Figures 2E and 4C). C-CMA^+^ bands were located in the terminal region of five chromosomes in *C. megalophylum*, on the short arms of pairs 4 and 7 and of the larger chromosome of the heteromorphic pair 1, and in the paracentromeric region (dot) of the long arms of pair 8 (Figures 2H and 4D). Only the bands observed in pairs 1 and 8 were C-CMA^+^/DAPI^+^.

All FISH assays with the 45S rDNA probe showed four terminal signals, preferentially on the short arms, except for a heteromorphism in *C. megalophylum*, where five chromosomes hybridized to the probe (Figures 3  and 4). Differences in signal sizes and positions were found. In *Cestrum capsulare,* hybridization sites were observed on pairs 6 and 8 (Figures 3A and 4A), and in *C. corymbosum* on pairs 7 and 8 (Figures 3B and 4B). In both cases, the 45S rDNA was associated with GC-rich bands. In *C. laevigatum,* signals were observed on pairs 3 and 7 (Figures 3C and 4C), but associated with C-CMA^+^/DAPI^+^ bands. In *C. megalophylum*, hybridization signals were observed on pairs 4 and 7, associated with GC-rich bands, besides the larger chromosome of the heteromorphic pair 1, associated with C-CMA^+^/DAPI^+^ bands (Figures 3D and 4D). FISH with the 5S rDNA probe showed hybridization signals always in the proximal region of pair 8, associated with GC-rich bands in *C. corymbosum* (Figures 3B and 4B) and associated with C-CMA^+^/DAPI^+^ bands in *C. laevigatum* and *C. megalophylum* (Figures 3C, 4C and Figures 3D and 4D, respectively). *Cestrum capsulare* showed a hybridization signal on pair 4, but also in the paracentromeric region of the largest chromosome (Figures 3A and 4A).

## Discussion

Cytogenetic analysis, performed for the first time in *C. capsulare, C. corymbosum, C. laevigatum* and *C. megalophylum*, showed a constant chromosome number (2*n* = 2*x* = 16), as previously described for *Cestrum* (Berg and Greilhuber, 1992, 1993a, 1993b; Fregonezi *et al.*, 2006). In addition to the numerical stability, other cytogenetic features were also constant, such as the predominance of meta- and submetacentric chromosomes, with pair 8 always being acrocentric, and the reticulate interphase nuclei (Berg and Greilhuber, 1992, 1993a, 1993b; Fregonezi *et al.*, 2006; Las Peñas *et al.*, 2006). Although there was a predominance of metacentrics and two submetacentrics for pair 8 in this study, some arm ratio values were very close to the limit between meta- and submetacentrics and submeta- and acrocentrics as defined by Guerra (1986) (see Figure 4). It is important to emphasize that the apparent constancy of chromosome number and karyotype morphology found in *Cestrum* was also observed in conventional cytogenetic studies of other Solanaceae, such as the genera *Capsicum* L. (Pozzobon *et al.*, 2006), *Solanum* L. (Bernardello and Anderson, 1990) and *Lycopersicum* Hill (Pillen *et al.*, 1996), all with 2*n* = 24.

Our results complement those obtained by Berg and Greilhuber (1992, 1993a, 1993b), Fregonezi *et al.* (2006) and Las Peñas *et al.* (2006), pointing out that there are some minor variations in the chromosome types regarding centromere position, chromosome size and karyotype symmetry. Las Peñas *et al.* (2006) proposed that an increase in genome size is accompanied by a small karyotype asymmetry, common in the tribe Cestreae. According to these authors, *Vestia* species are the most plesiomorphic within Cestreae, with the most symmetrical karyotypes and the smallest chromosomes. On the other hand, *Sessea* and *Cestrum* species are more apomorphic, with more asymmetrical karyotypes and larger chromosomes. Our results partially support the ideas of Las Peñas *et al.* (2006), once *C. capsulare* showed the most symmetrical karyotype and had the smallest haploid set size, while *C. megalophylum* presented the most asymmetrical karyotype and the largest haploid set size. This difference is sustained by taxonomic data, since *C. capsulare* was once classified into the genus *Sessea* (*Sessearegnellii* Taub.) and was later reclassified to *Cestrum* (Carvalho and Schnoor, 1997). However, chromosome banding (described below) showed that *C. capsulare* accumulated more repetitive DNA than the other three species, which could produce an increase in karyotype differences, including asymmetry.

Chromosome banding showed variation in the type, amount and distribution of heterochromatin among the four *Cestrum* species studied. However, we also found similarities, mainly with regard to the equilocal/equidistant and non- equilocal/equidistant distribution of bands (see Schweizer and Ehrendorfer, 1976; Schweizer and Loidl, 1987). C-CMA/DAPI banding allowed the identification of chromosomes and karyotypes, and the construction of idiograms, as previously demonstrated by Berg and Greilhuber (1992, 1993a, 1993b) and Fregonezi *et al.* (2006).

The physical maps defined three “domains” for heterochromatin occurrence in *Cestrum*. The first one was represented by the largest terminal C-CMA^+^ and C-CMA^+^/DAPI^+^ bands. The terminal C-CMA^+^ blocks were equilocal and equidistant, with interstitial dots on some heterologous chromosomes of *C. capsulare*. The second domain was represented by the largest interstitial/subterminal C-DAPI^+^ bands, which are equilocal and equidistant, with C-DAPI^+^ dots in *C. corymbosum* and less frequently in *C. laevigatum* and *C. megalophylum.* The third domain was represented by centromeric C-DAPI^+^ bands in *Cestrum capsulare* and *C. megalophylum*. The occurrence of these “domains” could be explained by the model of Schweizer and Loidl (1987), where different types of heterochromatin could begin preferentially in terminal regions and afterwards “contaminate” the interstitial regions of adjacent non-homologous chromosomes. Similar cases were reported in *Gibasis karwinskyana* (Roem. & Schult.) Rohw., Commelinaceae (Kenton, 1991) and some *Cestrum* species (Berg and Greilhuber, 1992, 1993a, 1993b; Fregonezi *et al.*, 2006). This kind of dispersion was found here in *C. corymbosum* and *C. megalophylum*, and was previously reported for *C. intermedium* and *C. strigilatum* (Fregonezi *et al.*, 2006).

The centromeric heterochromatin dispersion pattern detected by chromosome banding procedures can be considered of little importance in *Cestrum*, if compared with other plant groups, for example *Ophrys* L. (Orchidaceae). D'Emerico *et al.* (2005) studied seven species of *Ophrys* and found evident centromeric C-bands (Giemsa) in all the chromosomes. Another example of repetitive DNA accumulation in the centromeric regions of plants was published by Valárik *et al.* (2002). These authors isolated and cloned several repetitive DNA sequences of *Musa acuminata* L. (bananas) and, except for those associated with NORs, they appeared clustered in the centromeric regions of all chromosomes, as detected by FISH. These lines of evidence endorse the idea that there are specific positions in the chromosomes where arrays of repetitive DNA are accumulated, favored or tolerated. According to Flavell (1986), the selection process against or in favor of repetitive DNA accumulation can occur in differential ways, and it could also explain the different physical maps generated for *Cestrum*.

FISH with ribosomal probes showed a common pattern of 45S rDNA distribution in the four species studied here compared to other *Cestrum* species (Fregonezi *et al.*, 2006), since their hybridization sites were always located in the terminal regions of the chromosomes. These segments were found in different numbers but always in terminal regions, as in other genera of Solanaceae, such as *Capsicum* (Moscone *et al.*, 1995) and *Nicotiana* L. (Lim *et al.*, 2000). *Cestrum capsulare, C. corymbosum* and *C. laevigatum* showed four 45S rDNA sites, whereas *C. megalophylum* exhibited a heteromorphic pair with a terminal C-CMA^+^/NOR segment. NOR-associated heteromorphisms can happen due to non-equal crossing-over, non-reciprocal translocations or deletions. However, our data did not allow us to determine responsible event for these heteromorphisms, as well as if this event was fixed in the population or not, because samples of few individuals were collected in a restricted area. Different numbers of 45S rDNA sites have been described for *Cestrum*, varying from four to eight (Fregonezi *et al.*, 2006). Thus, events involving the amplification of rDNA repeats or an increase in the number of sites do not seem to affect the adaptive success of *Cestrum* species. Additionally, it is possible that other events are responsible for the dispersion of 45S rDNA repeats. Fregonezi *et al.* (2007) showed that a representative of the *Ty*3-*gypsy* family appears to be associated with NORs of *C. intermedium* and *C. strigilatum*, and that this retroelement could be involved in rDNA mobility among *Cestrum* species. Franz *et al.* (2000) suggested that in *Arabidopsis* the various types of transposable elements associated to NOR regions might favor rDNA mobility and affect both the evolution and the expression of rDNA loci.

The location of the 5S rDNA in paracentromeric regions of the long arm of pair 8 in *C. corymbosum, C. laevigatum* and *C. megalophylum* is in agreement with that described by Fregonezi *et al.* (2006) for other *Cestrum* species. Differences were found in *Cestrum capsulare*, where its chromosome position is the same, but on a different chromosome (pair 4). It is possible that a translocation has moved the 5S rDNA segment from pair 8 to pair 4, since the long arm of pair 8 of *C. capsulare* decreased in size and, according to measurements, this pair is not acrocentric as in other *Cestrum* species, but submetacentric. Another karyotype feature that attracted our attention was the differential association of 5S rDNA with heterochromatin. In this regard, we observed three different conditions: (i) lack of association with heterochromatin, as in *C. capsulare;* (ii) association with C-CMA^+^ segments, as found in *C. corymbosum*; and (iii) association with C-CMA^+^/DAPI^+^ segments, as found in *C. laevigatum* and *C. megalophylum*. The co-location of 5S rDNA and heterochromatin has already been described for other species. Xu and Earle (1996) showed that in *Lycopersicum* (Solanaceae) the 5S rDNA was associated with heterochromatic bands, and Cerbah *et al.* (1998) and Ruas *et al.* (2005) reported this association in several species of *Hypochoeris* L. (Asteraceae). The occurrence of paracentromeric 5S rDNA on the long arm of pair 8 is considered conserved in *Cestrum*, but the discovery in *C. capsulare*, which had been earlier ascribed to the genus *Sessea* (*S. regnellii*), can indicate two possibilities: (i) with the increase in the number of species studied, this location cannot be considered a good chromosome marker for the genus; or (ii) our results may be in contradiction to the junction of the genera *Cestrum* and *Sessea*, which was based on the diagnosis of fruit morphology, as proposed by Carvalho and Schnoor (1997). However, when we consider the general cytogenetic features of *C. capsulare*, including large chromosomes, a great number of heterochromatic blocks, equilocal distribution and number and location of 45S rDNA, *C. capsulare* seems to be well bounded.

In conclusion, the physical mapping generated after fluorescent chromosome banding and FISH with 45S and 5S rDNA, when compared with the maps generated for other *Cestrum* species, reinforces the idea that the karyotype changes in *Cestrum* resulted mainly from a great variation in the occurrence and distribution of different repetitive DNA segments, without major modifications in the karyotype formulae and chromosome size.
